# Poly[μ-aqua-aqua­{μ-6-eth­oxy-2-[(2-isonicotinoylhydrazinyl­idene)meth­yl]phenolato-κ^3^ *O*,*N*,*O*′}dioxidosodium­vanadate(V)]

**DOI:** 10.1107/S1600536811019106

**Published:** 2011-05-25

**Authors:** Hon Wee Wong, Kong Mun Lo, Seik Weng Ng

**Affiliations:** a Department of Chemistry, University of Malaya, 50603 Kuala Lumpur, Malaysia

## Abstract

The V^V^ atom in the polymeric title compound, [NaV(C_15_H_13_N_3_O_3_)O_2_(H_2_O)_2_]_*n*_, is *O*,*N*,*O*′-chelated by the Schiff base dianion and is five-coordinated in a trigonal–bipramidal coordination geometry. The oxide O atoms occupy the equatorial sites and one oxide O atom is connected to the Na^I^ atom. The ligand simultaneously *O*,*O*′-chelates to the water-coordinated Na^I^ atom; its coordination number is seven owing to an Na⋯N_pyrid­yl_ bond. The two independent formula units, which are disposed about a false center of inversion, are connected into a layer. Adjacent layers are consolidated into a three-dimensional network by O—H⋯O and O—H⋯N hydrogen bonds.

## Related literature

For the synthesis of isonicotinic acid (2-hy­droxy-3-eth­oxy­benzyl­idene)hydrazide, see: Georgieva & Gadjeva (2002 ▶). For related vanadates, see: Lippold *et al.* (2000 ▶); Plass *et al.* (2000 ▶); Plass & Yozgatli (2003 ▶).
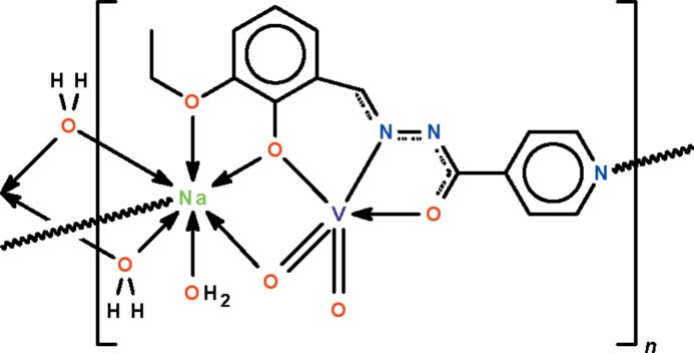


## Experimental

### 

#### Crystal data

[NaV(C_15_H_13_N_3_O_3_)O_2_(H_2_O)_2_]
*M* *_r_* = 425.25
Monoclinic, 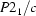

*a* = 19.1731 (2) Å
*b* = 14.6913 (2) Å
*c* = 13.2277 (2) Å
β = 106.4902 (6)°
*V* = 3572.69 (8) Å^3^
*Z* = 8
Mo *K*α radiation
μ = 0.62 mm^−1^
*T* = 100 K
0.40 × 0.40 × 0.40 mm

#### Data collection

Bruker SMART APEX diffractometer
Absorption correction: multi-scan (*SADABS*; Sheldrick, 1996 ▶) *T*_min_ = 0.789, *T*_max_ = 0.789
23057 measured reflections
8084 independent reflections
6785 reflections with *I* > 2σ(*I*)
*R*_int_ = 0.020

#### Refinement

*R*[*F* ^2^ > 2σ(*F* ^2^)] = 0.038
*wR*(*F* ^2^) = 0.120
*S* = 1.04
8084 reflections
489 parameters
H-atom parameters constrained
Δρ_max_ = 0.56 e Å^−3^
Δρ_min_ = −0.74 e Å^−3^

### 

Data collection: *APEX2* (Bruker, 2009 ▶); cell refinement: *SAINT* (Bruker, 2009 ▶); data reduction: *SAINT*; program(s) used to solve structure: *SHELXS97* (Sheldrick, 2008 ▶); program(s) used to refine structure: *SHELXL97* (Sheldrick, 2008 ▶); molecular graphics: *X-SEED* (Barbour, 2001 ▶); software used to prepare material for publication: *publCIF* (Westrip, 2010 ▶).

## Supplementary Material

Crystal structure: contains datablocks global, I. DOI: 10.1107/S1600536811019106/im2289sup1.cif

Structure factors: contains datablocks I. DOI: 10.1107/S1600536811019106/im2289Isup2.hkl

Additional supplementary materials:  crystallographic information; 3D view; checkCIF report

## Figures and Tables

**Table 1:** Hydrogen-bond geometry (Å, °)

*D*—H⋯*A*	*D*—H	H⋯*A*	*D*⋯*A*	*D*—H⋯*A*
O1*w*—H12⋯N2^i^	0.84	2.21	2.894 (2)	139
O2*w*—H21⋯N5^ii^	0.84	2.16	2.879 (2)	143
O3*w*—H32⋯O9	0.84	2.01	2.825 (2)	162
O3*w*—H31⋯O10^iii^	0.84	2.23	2.799 (2)	126
O4*w*—H41⋯O4	0.84	2.05	2.865 (2)	162
O4*w*—H42⋯O5^iv^	0.84	2.13	2.822 (2)	139

Symmetry codes: (i) 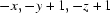; (ii) 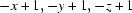; (iii) 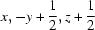; (iv) 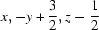.

**Fig. 1.**
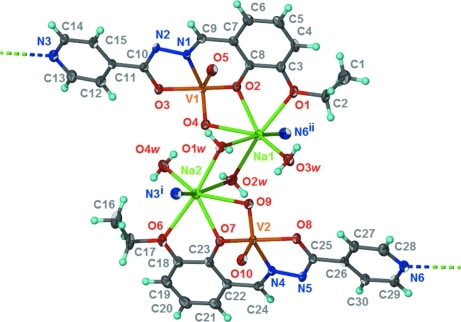
Thermal ellipsoid plot (Barbour, 2001) of a portion of the layer structure of [NaVO2(H2O)2(C15H13N3O2)]n at the 70% probability level; hydrogen atoms are drawn as spheres of arbitrary radius.

**Fig. 2.**
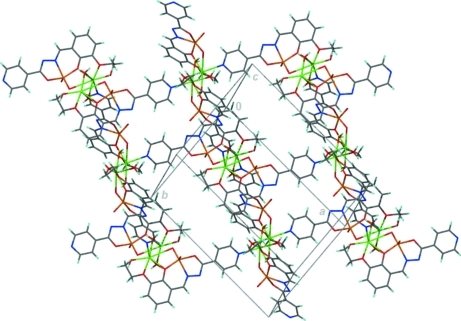
Packing diagram of the title structure.

## supplementary crystallographic information

### Comment

Schiff bases derived from condensation of isonicotinoyl hydrazide and *o*-vanillin type of aromatic aldehydes function as tridentate chelates towards a large number of metal ions. For the dioxovanadium(V) species in particularly, the dianionic nature of the deprotonated ligand requires a monocationic species to balance the charges. Isonicotinic acid (2-hydroxy-3-ethoxybenzylidene)hydrazide reacts with vanadyl(IV) sulfate in the presence of sodium acetate to yield the title vanadium(V) derivative, [NaVO_2_(H_2_O)_2_(C_15_H_13_N_3_O_2_)]*_n_* (Scheme I, Fig. 1). The V^V^ atom is *O*,*N*,*O*'-chelated by the Schiff base dianion, and is five-coordinate in a trigonal bipramidal geometry. The oxo O atoms occupy equatorial sites; one oxo O atom is connected to the Na^I^ atom. The ligand simultaneously *O*,*O*'-chelates to the water-coordinated Na^I^ atom; its coordination number is seven owing to an Na···N_pyridyl_ bond. The two independent formula units are connected into a layer. Adjacent layers are consolidated into a three-dimensional network by O–H···O and O–H···N hydrogen bonds (Table 1).

Dioxovanadates(V) based on similar Schiff bases whose negative charge is also balanced by a monovalent cation have been reported before (Lippold *et al.*, 2000; Plass *et al.*, 2000; Plass & Yozgatli; 2003).

### Experimental

The Schiff base was synthesized by using a literature procedure (Georgieva & Gadjeva, 2002) that involved condensing isonicotinic acid hydrazide (1 g, 7.5 mmol) and 3-ethoxysalicyldehyde (1.25 g, 7.5 mmol) in ethanol. The compound (1 g, 3.5 mmol), vanadyl(IV) sulfate (0.57 g, 3.5 mmol) and sodium acetate (0.3 g, 3.5 mmol) along with ethanol (100 ml) were were heated for 4 h. The solution was filtered and light brown crystals were obtained upon slow cooling of the solvent.

### Refinement

H atoms were placed in calculated positions (C—H 0.95 to 0.98, O—H 0.84 Å) and were included in the refinement in the riding model approximation, with *U*(H) set to 1.2 to 1.5 *U*(C,*O*). The water molecule that is connected to two Na atoms was treated as a methylene type whereas the water that is coordinated to only one Na atom was treated as a methyl type, but with the occupancy of one H atom being zero; H···H distances are a little longer than 2.0 Å.

### Crystal data

**Table:**

[NaV(C_15_H_13_N_3_O_3_)O_2_(H_2_O)_2_]	*F*(000) = 1744
*M**_r_* = 425.25	*D*_x_ = 1.581 Mg m^−^^3^
Monoclinic, *P*2_1_/*c*	Mo *K*α radiation, λ = 0.71073 Å
Hall symbol: -P 2ybc	Cell parameters from 9902 reflections
*a* = 19.1731 (2) Å	θ = 2.6–28.4°
*b* = 14.6913 (2) Å	µ = 0.62 mm^−^^1^
*c* = 13.2277 (2) Å	*T* = 100 K
β = 106.4902 (6)°	Block, dark brown
*V* = 3572.69 (8) Å^3^	0.40 × 0.40 × 0.40 mm
*Z* = 8	

### Data collection

**Table:**

Bruker SMART APEX diffractometer	8084 independent reflections
Radiation source: fine-focus sealed tube	6785 reflections with *I* > 2σ(*I*)
graphite	*R*_int_ = 0.020
ω scans	θ_max_ = 27.5°, θ_min_ = 2.2°
Absorption correction: multi-scan (*SADABS*; Sheldrick, 1996)	*h* = −24→24
*T*_min_ = 0.789, *T*_max_ = 0.789	*k* = −17→19
23057 measured reflections	*l* = −14→17

### Refinement

**Table:**

Refinement on *F*^2^	Primary atom site location: structure-invariant direct methods
Least-squares matrix: full	Secondary atom site location: difference Fourier map
*R*[*F*^2^ > 2σ(*F*^2^)] = 0.038	Hydrogen site location: inferred from neighbouring sites
*wR*(*F*^2^) = 0.120	H-atom parameters constrained
*S* = 1.04	*w* = 1/[σ^2^(*F*_o_^2^) + (0.0684*P*)^2^ + 2.7411*P*] where *P* = (*F*_o_^2^ + 2*F*_c_^2^)/3
8084 reflections	(Δ/σ)_max_ = 0.001
489 parameters	Δρ_max_ = 0.56 e Å^−^^3^
0 restraints	Δρ_min_ = −0.74 e Å^−^^3^

### Fractional atomic coordinates and isotropic or equivalent isotropic displacement parameters (Å^2^)

**Table:**

	*x*	*y*	*z*	*U*_iso_*/*U*_eq_	
V1	0.117273 (15)	0.64283 (2)	0.60824 (2)	0.01136 (9)	
V2	0.385383 (15)	0.35452 (2)	0.37870 (2)	0.01121 (9)	
Na1	0.26390 (4)	0.49475 (5)	0.64647 (6)	0.01554 (16)	
Na2	0.23922 (4)	0.50365 (5)	0.33801 (6)	0.01598 (17)	
O1	0.24513 (7)	0.43456 (10)	0.81471 (11)	0.0192 (3)	
O2	0.15035 (7)	0.53002 (9)	0.67556 (10)	0.0146 (3)	
O3	0.04859 (7)	0.70200 (9)	0.48708 (10)	0.0156 (3)	
O4	0.18565 (7)	0.65269 (9)	0.55605 (11)	0.0158 (3)	
O5	0.12649 (7)	0.71610 (9)	0.70224 (11)	0.0189 (3)	
O6	0.26740 (7)	0.57022 (10)	0.17087 (11)	0.0196 (3)	
O7	0.35533 (7)	0.47021 (9)	0.31652 (10)	0.0146 (3)	
O8	0.44973 (7)	0.28505 (9)	0.49566 (10)	0.0160 (3)	
O9	0.31480 (7)	0.34343 (9)	0.42565 (11)	0.0155 (3)	
O10	0.37743 (7)	0.28683 (9)	0.27927 (11)	0.0177 (3)	
O1w	0.19031 (7)	0.42958 (9)	0.47312 (11)	0.0168 (3)	
H11	0.1954	0.3728	0.4722	0.025*	
H12	0.1460	0.4416	0.4623	0.025*	
O2w	0.30731 (7)	0.57278 (9)	0.50975 (11)	0.0170 (3)	
H21	0.3523	0.5650	0.5209	0.026*	
H22	0.2990	0.6289	0.5098	0.026*	
O3w	0.32561 (8)	0.35904 (10)	0.64240 (12)	0.0233 (3)	
H31	0.3100	0.3187	0.6754	0.035*	
H32	0.3189	0.3426	0.5795	0.035*	
O4w	0.16471 (8)	0.63013 (10)	0.33428 (12)	0.0240 (3)	
H41	0.1705	0.6492	0.3960	0.036*	
H42	0.1758	0.6714	0.2978	0.036*	
N1	0.01291 (8)	0.58117 (10)	0.59125 (12)	0.0129 (3)	
N2	−0.04650 (8)	0.62497 (11)	0.52086 (12)	0.0131 (3)	
N3	−0.16486 (9)	0.88260 (12)	0.27671 (13)	0.0196 (3)	
N4	0.49119 (8)	0.41490 (11)	0.41014 (12)	0.0128 (3)	
N5	0.54804 (8)	0.36662 (11)	0.47984 (12)	0.0137 (3)	
N6	0.65617 (9)	0.10161 (12)	0.72044 (13)	0.0208 (4)	
C1	0.31672 (12)	0.41932 (18)	0.99966 (17)	0.0331 (5)	
H1A	0.3547	0.3823	1.0472	0.050*	
H1B	0.2725	0.4172	1.0227	0.050*	
H1C	0.3335	0.4824	1.0010	0.050*	
C2	0.30060 (11)	0.38249 (14)	0.88914 (16)	0.0215 (4)	
H2A	0.3457	0.3833	0.8669	0.026*	
H2B	0.2844	0.3185	0.8884	0.026*	
C3	0.17389 (10)	0.41942 (13)	0.81015 (15)	0.0157 (4)	
C4	0.14874 (11)	0.35585 (13)	0.86843 (16)	0.0200 (4)	
H4	0.1824	0.3210	0.9207	0.024*	
C5	0.07381 (12)	0.34254 (14)	0.85081 (16)	0.0225 (4)	
H5	0.0570	0.2979	0.8902	0.027*	
C6	0.02461 (11)	0.39376 (14)	0.77681 (16)	0.0198 (4)	
H6	−0.0261	0.3842	0.7650	0.024*	
C7	0.04927 (10)	0.46034 (13)	0.71855 (14)	0.0144 (4)	
C8	0.12410 (10)	0.47349 (12)	0.73408 (14)	0.0136 (3)	
C9	−0.00366 (10)	0.51195 (13)	0.64041 (14)	0.0146 (4)	
H9	−0.0533	0.4942	0.6242	0.018*	
C10	−0.02060 (9)	0.68851 (12)	0.47280 (14)	0.0136 (3)	
C11	−0.07164 (10)	0.75199 (13)	0.40071 (14)	0.0139 (4)	
C12	−0.04406 (11)	0.82798 (14)	0.36272 (16)	0.0207 (4)	
H12A	0.0069	0.8367	0.3772	0.025*	
C13	−0.09251 (11)	0.89082 (15)	0.30320 (17)	0.0232 (4)	
H13	−0.0729	0.9434	0.2796	0.028*	
C14	−0.19078 (10)	0.80815 (14)	0.31169 (15)	0.0175 (4)	
H14	−0.2420	0.8001	0.2932	0.021*	
C15	−0.14690 (10)	0.74169 (13)	0.37369 (15)	0.0162 (4)	
H15	−0.1680	0.6902	0.3971	0.019*	
C16	0.14547 (12)	0.57897 (18)	0.0608 (2)	0.0364 (6)	
H16A	0.1122	0.6113	0.0017	0.055*	
H16B	0.1280	0.5845	0.1234	0.055*	
H16C	0.1474	0.5146	0.0426	0.055*	
C17	0.21980 (12)	0.61956 (16)	0.08341 (18)	0.0295 (5)	
H17A	0.2378	0.6147	0.0205	0.035*	
H17B	0.2184	0.6847	0.1017	0.035*	
C18	0.34010 (10)	0.58845 (13)	0.19174 (15)	0.0171 (4)	
C19	0.36960 (12)	0.65790 (14)	0.14579 (17)	0.0227 (4)	
H19	0.3387	0.6959	0.0940	0.027*	
C20	0.44503 (12)	0.67204 (14)	0.17573 (17)	0.0238 (4)	
H20	0.4648	0.7208	0.1454	0.029*	
C21	0.49049 (11)	0.61638 (14)	0.24814 (16)	0.0204 (4)	
H21A	0.5416	0.6261	0.2674	0.025*	
C22	0.46124 (10)	0.54448 (13)	0.29418 (15)	0.0154 (4)	
C23	0.38582 (10)	0.53105 (12)	0.26846 (14)	0.0139 (4)	
C24	0.51098 (10)	0.48724 (13)	0.36962 (15)	0.0147 (4)	
H24	0.5610	0.5034	0.3908	0.018*	
C25	0.51939 (9)	0.29891 (13)	0.51815 (14)	0.0140 (4)	
C26	0.56794 (10)	0.23195 (13)	0.58868 (14)	0.0153 (4)	
C27	0.53878 (11)	0.15123 (14)	0.61331 (16)	0.0215 (4)	
H27	0.4882	0.1390	0.5863	0.026*	
C28	0.58465 (11)	0.08880 (15)	0.67801 (17)	0.0235 (4)	
H28	0.5641	0.0334	0.6932	0.028*	
C29	0.68389 (11)	0.17940 (14)	0.69501 (16)	0.0203 (4)	
H29	0.7346	0.1897	0.7231	0.024*	
C30	0.64247 (10)	0.24577 (14)	0.62976 (15)	0.0184 (4)	
H30	0.6647	0.2995	0.6136	0.022*	

### Atomic displacement parameters (Å^2^)

**Table:**

	*U*^11^	*U*^22^	*U*^33^	*U*^12^	*U*^13^	*U*^23^
V1	0.01057 (15)	0.01037 (16)	0.01223 (16)	−0.00005 (10)	0.00178 (12)	0.00022 (11)
V2	0.01082 (15)	0.01033 (16)	0.01139 (16)	−0.00039 (10)	0.00139 (12)	0.00012 (11)
Na1	0.0139 (3)	0.0157 (4)	0.0162 (4)	−0.0009 (3)	0.0030 (3)	−0.0007 (3)
Na2	0.0147 (3)	0.0154 (4)	0.0169 (4)	−0.0013 (3)	0.0029 (3)	−0.0016 (3)
O1	0.0161 (6)	0.0189 (7)	0.0204 (7)	0.0038 (5)	0.0016 (5)	0.0059 (5)
O2	0.0142 (6)	0.0132 (6)	0.0162 (6)	0.0016 (5)	0.0042 (5)	0.0041 (5)
O3	0.0115 (6)	0.0176 (7)	0.0166 (6)	0.0000 (5)	0.0022 (5)	0.0046 (5)
O4	0.0145 (6)	0.0144 (6)	0.0182 (7)	0.0016 (5)	0.0041 (5)	0.0029 (5)
O5	0.0191 (6)	0.0175 (7)	0.0180 (7)	−0.0003 (5)	0.0020 (5)	−0.0040 (5)
O6	0.0197 (7)	0.0181 (7)	0.0182 (7)	0.0040 (5)	0.0007 (5)	0.0049 (5)
O7	0.0151 (6)	0.0126 (6)	0.0161 (6)	0.0011 (5)	0.0042 (5)	0.0033 (5)
O8	0.0117 (6)	0.0178 (7)	0.0166 (6)	−0.0002 (5)	0.0011 (5)	0.0052 (5)
O9	0.0140 (6)	0.0151 (6)	0.0165 (6)	0.0013 (5)	0.0030 (5)	0.0021 (5)
O10	0.0174 (6)	0.0158 (7)	0.0183 (7)	0.0003 (5)	0.0025 (5)	−0.0028 (5)
O1w	0.0142 (6)	0.0146 (6)	0.0210 (7)	0.0001 (5)	0.0038 (5)	0.0004 (5)
O2w	0.0132 (6)	0.0154 (7)	0.0218 (7)	0.0003 (5)	0.0038 (5)	−0.0004 (5)
O3w	0.0310 (8)	0.0222 (8)	0.0175 (7)	0.0068 (6)	0.0084 (6)	0.0051 (6)
O4w	0.0311 (8)	0.0234 (8)	0.0179 (7)	0.0082 (6)	0.0076 (6)	0.0054 (6)
N1	0.0129 (7)	0.0126 (7)	0.0118 (7)	0.0025 (6)	0.0015 (6)	0.0004 (6)
N2	0.0103 (7)	0.0142 (7)	0.0130 (7)	0.0025 (6)	0.0004 (6)	0.0015 (6)
N3	0.0201 (8)	0.0194 (8)	0.0180 (8)	0.0022 (7)	0.0034 (6)	0.0038 (7)
N4	0.0127 (7)	0.0135 (7)	0.0110 (7)	0.0018 (6)	0.0014 (6)	0.0004 (6)
N5	0.0117 (7)	0.0145 (7)	0.0130 (7)	0.0018 (6)	0.0005 (6)	−0.0001 (6)
N6	0.0209 (8)	0.0222 (9)	0.0186 (8)	0.0049 (7)	0.0045 (7)	0.0049 (7)
C1	0.0264 (11)	0.0473 (15)	0.0218 (11)	0.0035 (10)	0.0005 (9)	−0.0052 (10)
C2	0.0192 (9)	0.0221 (10)	0.0194 (10)	0.0068 (8)	−0.0007 (7)	0.0029 (8)
C3	0.0164 (8)	0.0150 (9)	0.0144 (9)	0.0011 (7)	0.0023 (7)	−0.0010 (7)
C4	0.0246 (10)	0.0181 (10)	0.0147 (9)	0.0035 (7)	0.0014 (8)	0.0049 (7)
C5	0.0296 (11)	0.0192 (10)	0.0203 (10)	−0.0014 (8)	0.0094 (8)	0.0074 (8)
C6	0.0214 (9)	0.0191 (10)	0.0193 (9)	−0.0002 (8)	0.0066 (8)	0.0031 (8)
C7	0.0165 (8)	0.0135 (9)	0.0135 (8)	0.0004 (7)	0.0050 (7)	0.0004 (7)
C8	0.0181 (8)	0.0107 (8)	0.0120 (8)	0.0011 (7)	0.0042 (7)	0.0001 (6)
C9	0.0140 (8)	0.0149 (9)	0.0149 (9)	0.0007 (7)	0.0043 (7)	−0.0003 (7)
C10	0.0145 (8)	0.0134 (8)	0.0122 (8)	0.0019 (7)	0.0027 (7)	−0.0012 (7)
C11	0.0143 (8)	0.0153 (9)	0.0113 (8)	0.0022 (7)	0.0024 (7)	0.0011 (7)
C12	0.0160 (9)	0.0217 (10)	0.0238 (10)	−0.0010 (8)	0.0047 (8)	0.0068 (8)
C13	0.0198 (9)	0.0228 (10)	0.0252 (11)	−0.0015 (8)	0.0037 (8)	0.0096 (8)
C14	0.0133 (8)	0.0203 (10)	0.0170 (9)	0.0011 (7)	0.0011 (7)	0.0021 (7)
C15	0.0161 (8)	0.0151 (9)	0.0157 (9)	−0.0002 (7)	0.0017 (7)	0.0023 (7)
C16	0.0281 (11)	0.0395 (14)	0.0341 (13)	0.0074 (10)	−0.0032 (10)	0.0051 (11)
C17	0.0288 (11)	0.0266 (12)	0.0261 (11)	0.0099 (9)	−0.0036 (9)	0.0086 (9)
C18	0.0220 (9)	0.0140 (9)	0.0153 (9)	0.0024 (7)	0.0054 (7)	0.0003 (7)
C19	0.0328 (11)	0.0173 (10)	0.0187 (10)	0.0050 (8)	0.0086 (8)	0.0058 (8)
C20	0.0341 (11)	0.0173 (10)	0.0231 (11)	−0.0008 (8)	0.0129 (9)	0.0057 (8)
C21	0.0240 (10)	0.0182 (10)	0.0212 (10)	−0.0016 (8)	0.0098 (8)	0.0010 (8)
C22	0.0198 (9)	0.0118 (9)	0.0156 (9)	0.0008 (7)	0.0066 (7)	0.0002 (7)
C23	0.0187 (8)	0.0104 (8)	0.0126 (8)	0.0010 (7)	0.0043 (7)	−0.0003 (6)
C24	0.0135 (8)	0.0146 (9)	0.0167 (9)	−0.0020 (7)	0.0052 (7)	−0.0015 (7)
C25	0.0135 (8)	0.0160 (9)	0.0112 (8)	0.0013 (7)	0.0014 (6)	−0.0009 (7)
C26	0.0153 (8)	0.0172 (9)	0.0127 (8)	0.0017 (7)	0.0029 (7)	0.0016 (7)
C27	0.0154 (9)	0.0247 (11)	0.0228 (10)	0.0003 (8)	0.0026 (8)	0.0074 (8)
C28	0.0228 (10)	0.0217 (10)	0.0258 (11)	0.0008 (8)	0.0066 (8)	0.0093 (8)
C29	0.0171 (9)	0.0235 (10)	0.0183 (10)	0.0023 (8)	0.0018 (7)	0.0025 (8)
C30	0.0165 (9)	0.0198 (10)	0.0166 (9)	−0.0001 (7)	0.0012 (7)	0.0018 (8)

### Geometric parameters (Å, °)

**Table:**

V1—O5	1.6159 (14)	C1—C2	1.506 (3)
V1—O4	1.6523 (13)	C1—H1A	0.9800
V1—O2	1.9029 (13)	C1—H1B	0.9800
V1—O3	1.9648 (13)	C1—H1C	0.9800
V1—N1	2.1494 (15)	C2—H2A	0.9900
V2—O10	1.6211 (14)	C2—H2B	0.9900
V2—O9	1.6496 (13)	C3—C4	1.382 (3)
V2—O7	1.9048 (13)	C3—C8	1.418 (2)
V2—O8	1.9682 (13)	C4—C5	1.402 (3)
V2—N4	2.1439 (15)	C4—H4	0.9500
Na1—O3w	2.3268 (16)	C5—C6	1.375 (3)
Na1—O2	2.3732 (14)	C5—H5	0.9500
Na1—O2w	2.4764 (15)	C6—C7	1.408 (3)
Na1—O1	2.5136 (16)	C6—H6	0.9500
Na1—O1w	2.5157 (15)	C7—C8	1.404 (2)
Na1—N6^i^	2.5260 (18)	C7—C9	1.441 (2)
Na1—O4	2.8368 (15)	C9—H9	0.9500
Na2—O4w	2.3357 (16)	C10—C11	1.486 (2)
Na2—O7	2.3743 (15)	C11—C12	1.389 (3)
Na2—O1w	2.4918 (15)	C11—C15	1.393 (2)
Na2—O2w	2.4925 (15)	C12—C13	1.386 (3)
Na2—N3^ii^	2.5040 (18)	C12—H12A	0.9500
Na2—O6	2.6104 (16)	C13—H13	0.9500
Na2—O9	2.8338 (15)	C14—C15	1.394 (3)
O1—C3	1.368 (2)	C14—H14	0.9500
O1—C2	1.446 (2)	C15—H15	0.9500
O2—C8	1.328 (2)	C16—C17	1.495 (3)
O3—C10	1.301 (2)	C16—H16A	0.9800
O6—C18	1.368 (2)	C16—H16B	0.9800
O6—C17	1.448 (2)	C16—H16C	0.9800
O7—C23	1.325 (2)	C17—H17A	0.9900
O8—C25	1.299 (2)	C17—H17B	0.9900
O1w—H11	0.8400	C18—C19	1.388 (3)
O1w—H12	0.8400	C18—C23	1.416 (3)
O2w—H21	0.8400	C19—C20	1.402 (3)
O2w—H22	0.8400	C19—H19	0.9500
O3w—H31	0.8400	C20—C21	1.368 (3)
O3w—H32	0.8400	C20—H20	0.9500
O4w—H41	0.8400	C21—C22	1.412 (3)
O4w—H42	0.8400	C21—H21A	0.9500
N1—C9	1.294 (2)	C22—C23	1.402 (3)
N1—N2	1.406 (2)	C22—C24	1.440 (3)
N2—C10	1.304 (2)	C24—H24	0.9500
N3—C14	1.337 (3)	C25—C26	1.487 (2)
N3—C13	1.336 (3)	C26—C27	1.389 (3)
N3—Na2^iii^	2.5040 (18)	C26—C30	1.392 (2)
N4—C24	1.295 (2)	C27—C28	1.386 (3)
N4—N5	1.404 (2)	C27—H27	0.9500
N5—C25	1.306 (2)	C28—H28	0.9500
N6—C28	1.339 (3)	C29—C30	1.392 (3)
N6—C29	1.343 (3)	C29—H29	0.9500
N6—Na1^iv^	2.5260 (18)	C30—H30	0.9500
			
O5—V1—O4	109.68 (7)	C25—N5—N4	107.82 (14)
O5—V1—O2	105.72 (7)	C28—N6—C29	116.46 (17)
O4—V1—O2	94.20 (6)	C28—N6—Na1^iv^	123.45 (14)
O5—V1—O3	103.57 (7)	C29—N6—Na1^iv^	119.90 (13)
O4—V1—O3	92.75 (6)	C2—C1—H1A	109.5
O2—V1—O3	145.51 (6)	C2—C1—H1B	109.5
O5—V1—N1	104.86 (7)	H1A—C1—H1B	109.5
O4—V1—N1	144.96 (6)	C2—C1—H1C	109.5
O2—V1—N1	81.87 (6)	H1A—C1—H1C	109.5
O3—V1—N1	73.30 (6)	H1B—C1—H1C	109.5
O10—V2—O9	110.28 (7)	O1—C2—C1	112.30 (17)
O10—V2—O7	104.46 (6)	O1—C2—H2A	109.1
O9—V2—O7	94.06 (6)	C1—C2—H2A	109.1
O10—V2—O8	102.31 (6)	O1—C2—H2B	109.1
O9—V2—O8	92.93 (6)	C1—C2—H2B	109.1
O7—V2—O8	147.93 (6)	H2A—C2—H2B	107.9
O10—V2—N4	106.09 (6)	O1—C3—C4	125.96 (17)
O9—V2—N4	143.14 (6)	O1—C3—C8	113.76 (16)
O7—V2—N4	82.42 (6)	C4—C3—C8	120.22 (17)
O8—V2—N4	73.42 (6)	C3—C4—C5	120.31 (17)
O3w—Na1—O2	133.37 (6)	C3—C4—H4	119.8
O3w—Na1—O2w	95.81 (6)	C5—C4—H4	119.8
O2—Na1—O2w	122.09 (5)	C6—C5—C4	120.37 (18)
O3w—Na1—O1	85.44 (5)	C6—C5—H5	119.8
O2—Na1—O1	64.20 (5)	C4—C5—H5	119.8
O2w—Na1—O1	166.33 (5)	C5—C6—C7	120.06 (18)
O3w—Na1—O1w	79.05 (5)	C5—C6—H6	120.0
O2—Na1—O1w	85.86 (5)	C7—C6—H6	120.0
O2w—Na1—O1w	74.23 (5)	C8—C7—C6	120.24 (17)
O1—Na1—O1w	119.27 (5)	C8—C7—C9	121.00 (17)
O3w—Na1—N6^i^	109.68 (6)	C6—C7—C9	118.72 (17)
O2—Na1—N6^i^	98.88 (6)	O2—C8—C7	122.78 (16)
O2w—Na1—N6^i^	87.60 (6)	O2—C8—C3	118.29 (16)
O1—Na1—N6^i^	79.20 (6)	C7—C8—C3	118.76 (17)
O1w—Na1—N6^i^	160.75 (6)	N1—C9—C7	123.39 (17)
O3w—Na1—O4	154.51 (6)	N1—C9—H9	118.3
O2—Na1—O4	59.33 (4)	C7—C9—H9	118.3
O2w—Na1—O4	63.93 (4)	O3—C10—N2	123.49 (16)
O1—Na1—O4	118.01 (5)	O3—C10—C11	117.15 (16)
O1w—Na1—O4	80.46 (5)	N2—C10—C11	119.31 (16)
N6^i^—Na1—O4	85.94 (5)	C12—C11—C15	117.88 (17)
O4w—Na2—O7	138.65 (6)	C12—C11—C10	119.20 (16)
O4w—Na2—O1w	90.58 (5)	C15—C11—C10	122.83 (17)
O7—Na2—O1w	123.51 (5)	C13—C12—C11	118.59 (18)
O4w—Na2—O2w	81.61 (5)	C13—C12—H12A	120.7
O7—Na2—O2w	85.82 (5)	C11—C12—H12A	120.7
O1w—Na2—O2w	74.38 (5)	N3—C13—C12	124.57 (19)
O4w—Na2—N3^ii^	108.21 (6)	N3—C13—H13	117.7
O7—Na2—N3^ii^	99.93 (6)	C12—C13—H13	117.7
O1w—Na2—N3^ii^	81.90 (5)	N3—C14—C15	123.72 (17)
O2w—Na2—N3^ii^	154.50 (6)	N3—C14—H14	118.1
O4w—Na2—O6	87.50 (5)	C15—C14—H14	118.1
O7—Na2—O6	63.23 (5)	C11—C15—C14	118.90 (18)
O1w—Na2—O6	168.97 (5)	C11—C15—H15	120.5
O2w—Na2—O6	116.01 (5)	C14—C15—H15	120.5
N3^ii^—Na2—O6	88.39 (6)	C17—C16—H16A	109.5
O4w—Na2—O9	155.24 (6)	C17—C16—H16B	109.5
O7—Na2—O9	59.28 (4)	H16A—C16—H16B	109.5
O1w—Na2—O9	66.21 (4)	C17—C16—H16C	109.5
O2w—Na2—O9	83.74 (5)	H16A—C16—H16C	109.5
N3^ii^—Na2—O9	78.17 (5)	H16B—C16—H16C	109.5
O6—Na2—O9	116.93 (5)	O6—C17—C16	107.99 (18)
C3—O1—C2	118.43 (15)	O6—C17—H17A	110.1
C3—O1—Na1	114.22 (11)	C16—C17—H17A	110.1
C2—O1—Na1	121.76 (12)	O6—C17—H17B	110.1
C8—O2—V1	133.34 (12)	C16—C17—H17B	110.1
C8—O2—Na1	118.35 (11)	H17A—C17—H17B	108.4
V1—O2—Na1	108.27 (6)	O6—C18—C19	125.03 (18)
C10—O3—V1	118.08 (11)	O6—C18—C23	114.58 (17)
V1—O4—Na1	98.02 (6)	C19—C18—C23	120.37 (18)
C18—O6—C17	115.98 (16)	C18—C19—C20	120.07 (19)
C18—O6—Na2	110.26 (11)	C18—C19—H19	120.0
C17—O6—Na2	128.99 (13)	C20—C19—H19	120.0
C23—O7—V2	133.52 (12)	C21—C20—C19	120.69 (19)
C23—O7—Na2	118.30 (11)	C21—C20—H20	119.7
V2—O7—Na2	108.17 (6)	C19—C20—H20	119.7
C25—O8—V2	118.22 (11)	C20—C21—C22	119.78 (19)
V2—O9—Na2	98.21 (6)	C20—C21—H21A	120.1
Na2—O1w—Na1	105.05 (5)	C22—C21—H21A	120.1
Na2—O1w—H11	110.7	C23—C22—C21	120.60 (17)
Na1—O1w—H11	110.7	C23—C22—C24	121.37 (17)
Na2—O1w—H12	110.7	C21—C22—C24	118.02 (17)
Na1—O1w—H12	110.7	O7—C23—C22	122.92 (16)
H11—O1w—H12	108.8	O7—C23—C18	118.56 (16)
Na1—O2w—Na2	106.22 (5)	C22—C23—C18	118.43 (17)
Na1—O2w—H21	110.5	N4—C24—C22	123.52 (17)
Na2—O2w—H21	110.5	N4—C24—H24	118.2
Na1—O2w—H22	110.5	C22—C24—H24	118.2
Na2—O2w—H22	110.5	O8—C25—N5	123.27 (16)
H21—O2w—H22	108.7	O8—C25—C26	117.36 (16)
Na1—O3w—H31	109.5	N5—C25—C26	119.34 (16)
Na1—O3w—H32	109.5	C27—C26—C30	118.03 (17)
H31—O3w—H32	109.5	C27—C26—C25	119.35 (17)
Na2—O4w—H41	109.5	C30—C26—C25	122.60 (17)
Na2—O4w—H42	109.5	C26—C27—C28	118.89 (18)
H41—O4w—H42	109.5	C26—C27—H27	120.6
C9—N1—N2	115.09 (15)	C28—C27—H27	120.6
C9—N1—V1	129.24 (12)	N6—C28—C27	124.10 (19)
N2—N1—V1	115.57 (11)	N6—C28—H28	118.0
C10—N2—N1	107.54 (14)	C27—C28—H28	118.0
C14—N3—C13	116.31 (17)	N6—C29—C30	123.70 (18)
C14—N3—Na2^iii^	125.95 (13)	N6—C29—H29	118.2
C13—N3—Na2^iii^	117.70 (13)	C30—C29—H29	118.2
C24—N4—N5	114.99 (15)	C26—C30—C29	118.79 (18)
C24—N4—V2	129.18 (12)	C26—C30—H30	120.6
N5—N4—V2	115.71 (11)	C29—C30—H30	120.6
			
O3w—Na1—O1—C3	−116.11 (13)	O6—Na2—O2w—Na1	178.74 (5)
O2—Na1—O1—C3	27.38 (12)	O9—Na2—O2w—Na1	−64.29 (5)
O2w—Na1—O1—C3	148.0 (2)	O5—V1—N1—C9	−87.20 (17)
O1w—Na1—O1—C3	−41.36 (14)	O4—V1—N1—C9	102.71 (18)
N6^i^—Na1—O1—C3	132.84 (13)	O2—V1—N1—C9	17.00 (16)
O4—Na1—O1—C3	53.39 (13)	O3—V1—N1—C9	172.79 (17)
O3w—Na1—O1—C2	37.07 (14)	O5—V1—N1—N2	89.02 (13)
O2—Na1—O1—C2	−179.45 (15)	O4—V1—N1—N2	−81.06 (16)
O2w—Na1—O1—C2	−58.8 (3)	O2—V1—N1—N2	−166.78 (12)
O1w—Na1—O1—C2	111.82 (14)	O3—V1—N1—N2	−10.99 (11)
N6^i^—Na1—O1—C2	−73.98 (14)	C9—N1—N2—C10	−175.67 (16)
O4—Na1—O1—C2	−153.43 (13)	V1—N1—N2—C10	7.56 (18)
O5—V1—O2—C8	69.85 (17)	O10—V2—N4—C24	87.21 (17)
O4—V1—O2—C8	−178.35 (16)	O9—V2—N4—C24	−102.38 (18)
O3—V1—O2—C8	−77.29 (19)	O7—V2—N4—C24	−15.73 (16)
N1—V1—O2—C8	−33.38 (16)	O8—V2—N4—C24	−174.41 (18)
O5—V1—O2—Na1	−107.86 (7)	O10—V2—N4—N5	−88.64 (13)
O4—V1—O2—Na1	3.95 (7)	O9—V2—N4—N5	81.78 (16)
O3—V1—O2—Na1	105.01 (10)	O7—V2—N4—N5	168.43 (13)
N1—V1—O2—Na1	148.91 (7)	O8—V2—N4—N5	9.74 (11)
O3w—Na1—O2—C8	27.16 (16)	C24—N4—N5—C25	176.69 (16)
O2w—Na1—O2—C8	166.35 (12)	V2—N4—N5—C25	−6.87 (18)
O1—Na1—O2—C8	−27.53 (12)	C3—O1—C2—C1	−79.2 (2)
O1w—Na1—O2—C8	97.88 (13)	Na1—O1—C2—C1	128.68 (16)
N6^i^—Na1—O2—C8	−100.90 (13)	C2—O1—C3—C4	−2.3 (3)
O4—Na1—O2—C8	179.23 (14)	Na1—O1—C3—C4	151.87 (17)
O3w—Na1—O2—V1	−154.74 (7)	C2—O1—C3—C8	−179.53 (16)
O2w—Na1—O2—V1	−15.55 (9)	Na1—O1—C3—C8	−25.40 (19)
O1—Na1—O2—V1	150.57 (8)	O1—C3—C4—C5	−175.34 (19)
O1w—Na1—O2—V1	−84.02 (6)	C8—C3—C4—C5	1.8 (3)
N6^i^—Na1—O2—V1	77.20 (7)	C3—C4—C5—C6	−1.2 (3)
O4—Na1—O2—V1	−2.67 (5)	C4—C5—C6—C7	−0.3 (3)
O5—V1—O3—C10	−89.13 (14)	C5—C6—C7—C8	1.3 (3)
O4—V1—O3—C10	159.87 (13)	C5—C6—C7—C9	179.06 (19)
O2—V1—O3—C10	58.37 (17)	V1—O2—C8—C7	33.3 (3)
N1—V1—O3—C10	12.58 (13)	Na1—O2—C8—C7	−149.21 (14)
O5—V1—O4—Na1	105.19 (7)	V1—O2—C8—C3	−151.49 (14)
O2—V1—O4—Na1	−3.17 (6)	Na1—O2—C8—C3	26.0 (2)
O3—V1—O4—Na1	−149.36 (5)	C6—C7—C8—O2	174.43 (17)
N1—V1—O4—Na1	−85.00 (11)	C9—C7—C8—O2	−3.2 (3)
O3w—Na1—O4—V1	130.65 (12)	C6—C7—C8—C3	−0.8 (3)
O2—Na1—O4—V1	2.94 (5)	C9—C7—C8—C3	−178.48 (17)
O2w—Na1—O4—V1	170.81 (7)	O1—C3—C8—O2	1.3 (2)
O1—Na1—O4—V1	−24.39 (8)	C4—C3—C8—O2	−176.18 (17)
O1w—Na1—O4—V1	93.78 (6)	O1—C3—C8—C7	176.70 (16)
N6^i^—Na1—O4—V1	−99.88 (7)	C4—C3—C8—C7	−0.7 (3)
O4w—Na2—O6—C18	118.39 (12)	N2—N1—C9—C7	−177.61 (16)
O7—Na2—O6—C18	−31.34 (11)	V1—N1—C9—C7	−1.4 (3)
O1w—Na2—O6—C18	−161.4 (3)	C8—C7—C9—N1	−10.3 (3)
O2w—Na2—O6—C18	38.97 (13)	C6—C7—C9—N1	172.02 (18)
N3^ii^—Na2—O6—C18	−133.30 (12)	V1—O3—C10—N2	−13.9 (2)
O9—Na2—O6—C18	−57.44 (13)	V1—O3—C10—C11	163.67 (12)
O4w—Na2—O6—C17	−35.70 (17)	N1—N2—C10—O3	3.3 (2)
O7—Na2—O6—C17	174.58 (17)	N1—N2—C10—C11	−174.22 (15)
O1w—Na2—O6—C17	44.5 (4)	O3—C10—C11—C12	−7.8 (3)
O2w—Na2—O6—C17	−115.11 (16)	N2—C10—C11—C12	169.91 (18)
N3^ii^—Na2—O6—C17	72.61 (17)	O3—C10—C11—C15	175.95 (17)
O9—Na2—O6—C17	148.47 (16)	N2—C10—C11—C15	−6.4 (3)
O10—V2—O7—C23	−73.90 (17)	C15—C11—C12—C13	2.1 (3)
O9—V2—O7—C23	173.94 (16)	C10—C11—C12—C13	−174.36 (18)
O8—V2—O7—C23	71.8 (2)	C14—N3—C13—C12	0.4 (3)
N4—V2—O7—C23	30.84 (16)	Na2^iii^—N3—C13—C12	−177.51 (17)
O10—V2—O7—Na2	107.11 (7)	C11—C12—C13—N3	−1.9 (3)
O9—V2—O7—Na2	−5.05 (7)	C13—N3—C14—C15	0.9 (3)
O8—V2—O7—Na2	−107.14 (10)	Na2^iii^—N3—C14—C15	178.59 (14)
N4—V2—O7—Na2	−148.15 (7)	C12—C11—C15—C14	−1.0 (3)
O4w—Na2—O7—C23	−18.30 (17)	C10—C11—C15—C14	175.36 (17)
O1w—Na2—O7—C23	−158.72 (12)	N3—C14—C15—C11	−0.6 (3)
O2w—Na2—O7—C23	−90.57 (13)	C18—O6—C17—C16	169.99 (18)
N3^ii^—Na2—O7—C23	114.48 (13)	Na2—O6—C17—C16	−37.1 (3)
O6—Na2—O7—C23	31.39 (12)	C17—O6—C18—C19	9.2 (3)
O9—Na2—O7—C23	−175.76 (14)	Na2—O6—C18—C19	−148.61 (17)
O4w—Na2—O7—V2	160.87 (7)	C17—O6—C18—C23	−172.29 (17)
O1w—Na2—O7—V2	20.45 (9)	Na2—O6—C18—C23	29.91 (19)
O2w—Na2—O7—V2	88.60 (6)	O6—C18—C19—C20	177.99 (19)
N3^ii^—Na2—O7—V2	−66.35 (7)	C23—C18—C19—C20	−0.4 (3)
O6—Na2—O7—V2	−149.44 (8)	C18—C19—C20—C21	1.8 (3)
O9—Na2—O7—V2	3.41 (5)	C19—C20—C21—C22	−0.7 (3)
O10—V2—O8—C25	92.32 (14)	C20—C21—C22—C23	−1.7 (3)
O9—V2—O8—C25	−156.19 (14)	C20—C21—C22—C24	179.70 (19)
O7—V2—O8—C25	−53.77 (18)	V2—O7—C23—C22	−31.7 (3)
N4—V2—O8—C25	−11.03 (13)	Na2—O7—C23—C22	147.24 (15)
O10—V2—O9—Na2	−102.99 (7)	V2—O7—C23—C18	151.86 (14)
O7—V2—O9—Na2	4.06 (6)	Na2—O7—C23—C18	−29.2 (2)
O8—V2—O9—Na2	152.74 (5)	C21—C22—C23—O7	−173.52 (17)
N4—V2—O9—Na2	86.83 (10)	C24—C22—C23—O7	5.0 (3)
O4w—Na2—O9—V2	−146.59 (12)	C21—C22—C23—C18	3.0 (3)
O7—Na2—O9—V2	−3.78 (5)	C24—C22—C23—C18	−178.50 (17)
O1w—Na2—O9—V2	−168.28 (7)	O6—C18—C23—O7	−3.8 (3)
O2w—Na2—O9—V2	−92.62 (6)	C19—C18—C23—O7	174.75 (18)
N3^ii^—Na2—O9—V2	105.45 (7)	O6—C18—C23—C22	179.53 (16)
O6—Na2—O9—V2	23.42 (8)	C19—C18—C23—C22	−1.9 (3)
O4w—Na2—O1w—Na1	−83.78 (6)	N5—N4—C24—C22	178.13 (17)
O7—Na2—O1w—Na1	71.33 (7)	V2—N4—C24—C22	2.3 (3)
O2w—Na2—O1w—Na1	−2.65 (5)	C23—C22—C24—N4	7.5 (3)
N3^ii^—Na2—O1w—Na1	167.91 (6)	C21—C22—C24—N4	−173.93 (18)
O6—Na2—O1w—Na1	−163.7 (3)	V2—O8—C25—N5	12.0 (2)
O9—Na2—O1w—Na1	87.32 (5)	V2—O8—C25—C26	−166.18 (12)
O3w—Na1—O1w—Na2	−96.64 (6)	N4—N5—C25—O8	−2.6 (2)
O2—Na1—O1w—Na2	127.70 (6)	N4—N5—C25—C26	175.49 (15)
O2w—Na1—O1w—Na2	2.67 (5)	O8—C25—C26—C27	10.4 (3)
O1—Na1—O1w—Na2	−175.03 (5)	N5—C25—C26—C27	−167.87 (18)
N6^i^—Na1—O1w—Na2	22.5 (2)	O8—C25—C26—C30	−171.11 (18)
O4—Na1—O1w—Na2	68.12 (5)	N5—C25—C26—C30	10.6 (3)
O3w—Na1—O2w—Na2	74.19 (6)	C30—C26—C27—C28	0.6 (3)
O2—Na1—O2w—Na2	−77.29 (7)	C25—C26—C27—C28	179.18 (19)
O1—Na1—O2w—Na2	168.8 (2)	C29—N6—C28—C27	−1.8 (3)
O1w—Na1—O2w—Na2	−2.69 (5)	Na1^iv^—N6—C28—C27	173.16 (17)
N6^i^—Na1—O2w—Na2	−176.27 (6)	C26—C27—C28—N6	1.0 (3)
O4—Na1—O2w—Na2	−89.62 (5)	C28—N6—C29—C30	1.0 (3)
O4w—Na2—O2w—Na1	95.68 (6)	Na1^iv^—N6—C29—C30	−174.16 (15)
O7—Na2—O2w—Na1	−123.81 (6)	C27—C26—C30—C29	−1.3 (3)
O1w—Na2—O2w—Na1	2.71 (5)	C25—C26—C30—C29	−179.86 (18)
N3^ii^—Na2—O2w—Na1	−19.44 (16)	N6—C29—C30—C26	0.5 (3)

Symmetry codes: (i) −*x*+1, *y*+1/2, −*z*+3/2; (ii) −*x*, *y*−1/2, −*z*+1/2; (iii) −*x*, *y*+1/2, −*z*+1/2; (iv) −*x*+1, *y*−1/2, −*z*+3/2.

### Hydrogen-bond geometry (Å, °)

**Table:**

*D*—H···*A*	*D*—H	H···*A*	*D*···*A*	*D*—H···*A*
O1w—H12···N2^v^	0.84	2.21	2.894 (2)	139
O2w—H21···N5^vi^	0.84	2.16	2.879 (2)	143
O3w—H32···O9	0.84	2.01	2.825 (2)	162
O3w—H31···O10^vii^	0.84	2.23	2.799 (2)	126
O4w—H41···O4	0.84	2.05	2.865 (2)	162
O4w—H42···O5^viii^	0.84	2.13	2.822 (2)	139

Symmetry codes: (v) −*x*, −*y*+1, −*z*+1; (vi) −*x*+1, −*y*+1, −*z*+1; (vii) *x*, −*y*+1/2, *z*+1/2; (viii) *x*, −*y*+3/2, *z*−1/2.
